# The Legend of the Buffalo Chest

**DOI:** 10.1016/j.chest.2021.06.043

**Published:** 2021-06-30

**Authors:** Marielle M.J. Blacha, Illaa Smesseim, Ivo van der Lee, Joost G. van den Aardweg, Marcus J. Schultz, Marja L.J. Kik, Linda van Sonsbeek, Bernadette S. de Bakker, Richard W. Light

**Affiliations:** a Department of Intensive Care, Amsterdam University Medical Centers, AMC, University of Amsterdam, Amsterdam, The Netherlands; b Laboratory of Experimental Intensive Care and Anesthesiology (L·E·I·C·A), Amsterdam University Medical Centers, AMC, University of Amsterdam, Amsterdam, The Netherlands; c Department of Medical Biology, Section Clinical Anatomy & Embryology, Amsterdam University Medical Centers, AMC, University of Amsterdam, Amsterdam, The Netherlands; d Department of Respiratory Medicine and Clinical Physiology, Amsterdam University Medical Centers, VU Medical University, Amsterdam, The Netherlands; e Department of Pulmonary Medicine, Spaarne Gasthuis, Haarlem, The Netherlands; f Mahidol-Oxford Tropical Medicine Research Unit (MORU), Mahidol University, Bangkok, Thailand; g Mahidol-Oxford Tropical Medicine Research Unit (MORU), University of Oxford, Oxford, United Kingdom; h Department of Biomedical Health Sciences, Pathology Division, Veterinary Medicine, Utrecht University, Utrecht, The Netherlands; i Department of Veterinary Medicine, Rotterdam Zoo Blijdorp, Rotterdam, The Netherlands; j Division of Allergy, Pulmonary and Critical Care Medicine, Vanderbilt University, Nashville, TN

**Keywords:** buffalo chest, pleural diseases, pneumothorax

## Abstract

**Background:**

The “buffalo chest” is a condition in which a simultaneous bilateral pneumothorax occurs due to a communication of both pleural cavities caused by an iatrogenic or idiopathic fenestration of the mediastinum. This rare condition is known by many clinicians because of a particular anecdote which stated that Native Americans could kill a North American bison with a single arrow in the chest by creating a simultaneous bilateral pneumothorax, due to the animal’s peculiar anatomy in which there is one contiguous pleural space due to an incomplete mediastinum.

**Research Question:**

What evidence is there for the existence of buffalo chest?

**Study Design and Methods:**

The term “buffalo chest” and its anecdote were first mentioned in a ‘‘personal communication’’ by a veterinarian in the *Annals of Surgery* in 1984. A mixed method research was performed on buffalo chest and its etiology. A total of 47 cases of buffalo chest were identified in humans.

**Results:**

This study found that all authors were referring to the article from 1984 or to each other. Evidence was found for interpleural communications in other mammal species, but no literature on the anatomy of the mediastinum of the bison was found. The main reason for this research was fact-checking the origin of the anecdote and search for evidence for the existence of buffalo chest. Autopsies were performed on eight bison, and four indeed were found to have had interpleural communications.

**Interpretation:**

We hypothesize that humans can also have interpleural fenestrations, which can be diagnosed when a pneumothorax occurs.

## Graphical Abstract

**Figure:**
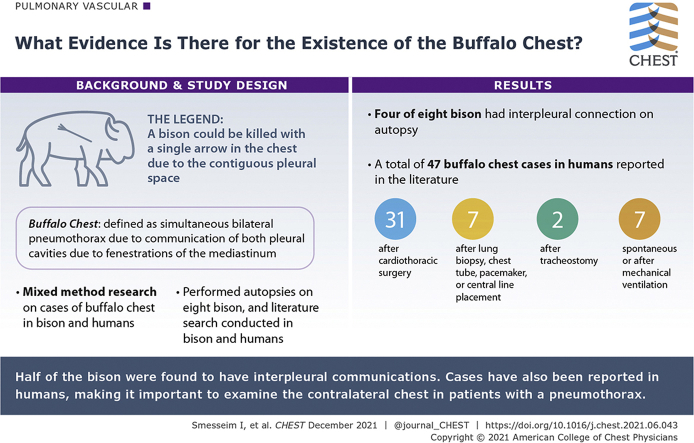

**Take-home Points**

**Study Question:** Is the “buffalo chest” an existing entity in North American bison?

**Results:** In autopsies on eight bison, four of the animals were found to have interpleural communications.

**Interpretation:** Because humans share many similarities with other mammals in embryologic development, it is important to always examine the contralateral chest when a patient presents with a pneumothorax as a bilateral pneumothorax that is overlooked can be fatal because of the risk of a bilateral tension pneumothorax.

In February 2018, *The New England Journal of Medicine* published a review article entitled “Pleural disease” by David Feller-Kopman and Richard W. Light.^1^ As residents in pulmonology, we read this article during our Journal Club and were intrigued by a particular clinical condition that was mentioned in the article: the buffalo chest. Buffalo chest is a potentially life-threatening condition in which a simultaneous bilateral pneumothorax occurs due to a communication of both pleural cavities caused by iatrogenic or idiopathic fenestrations of the mediastinum.

This condition is known by many clinicians because of a particular anecdote which stated that Native Americans could kill North American bison (*Bison bison*) with a single arrow or gunshot in the chest by creating a bilateral pneumothorax. Even though it is stated that the buffalo chest is a very rare condition,^2^ we were surprised that almost every pulmonologist, surgeon, and radiologist of whom we requested information remembered a specific case of a patient with a buffalo chest.

Schorlemmer et al^3^ were the first authors who introduced the term buffalo chest in 1984. They reported a case of a simultaneous bilateral pneumothorax following a subclavian venous catheterization in a patient who had undergone a median sternotomy causing a communication of both pleural cavities. In their article, they referred to a “personal communication” by a veterinarian named Dr Kenneth Throlson. He had told them that the interpleural communication in their patient could be compared with an anatomical entity he found on occasion in the North American bison. He thought this was also known by Native Americans who used this knowledge for hunting bison. With a single chest wound they killed their prey by causing a bilateral pneumothorax that resulted in a rapid death.

## Methods

We performed a literature search on the buffalo chest in bison and its etiology. MEDLINE, PubMed, and Google Scholar were searched for articles. The following key words and Medical Subject Heading terms were used: buffalo chest, pleuro-pleural communication, bilateral pneumothorax, simultaneous bilateral pneumothorax, pneumothorax in bison, mediastinum in bison, anatomy of bison, pneumothorax in animals, anatomy of mediastinum, embryological development of the lungs, and embryologic development of pleural cavity. Reference lists of relevant papers were checked. Eligible papers were reviewed by two researchers (M. M. J. B. and I. S.), and data were extracted on patient demographic characteristics.

We identified a total of 47 cases of patients with a buffalo chest in 34 different case reports.^3^, ^4^, ^5^, ^6^, ^7^, ^8^, ^9^, ^10^, ^11^, ^12^, ^13^, ^14^, ^15^, ^16^, ^17^, ^18^, ^19^, ^20^, ^21^, ^22^, ^23^, ^24^, ^25^, ^26^, ^27^, ^28^, ^29^, ^30^, ^31^, ^32^, ^33^, ^34^, ^35^, ^36^ Most of these were case reports that mentioned patients who had undergone previous (cardio)thoracic surgery^3^, ^4^, ^5^, ^6^, ^7^, ^8^, ^9^, ^10^, ^11^, ^12^, ^13^, ^14^, ^15^, ^16^, ^17^, ^18^, ^19^, ^20^, ^21^ (Table 1). It is known that (cardio)thoracic surgery can result in bilateral pleural fenestrations that can become a persistent interpleural communication in the mediastinum. Primary or secondary unilateral pneumothoraces could therefore result in airflow to the contralateral pleural cavity through the interpleural communication and subsequently generate a bilateral pneumothorax.

**Table 1:** Simultaneous Bilateral Pneumothorax in Cases With Iatrogenic Communication Between Both Pleural Cavities

Author	No. of Cases	Patient Sex	Previous Intrathoracic Surgery
Grathwohl and Derdak^4^	1	Male	Following right-sided pneumonectomy
Ikezoe et al^5^	1	Male	Extended thymectomy by median sternotomy
Schorlemmer et al^3^	1	Male	Right infraclavicular subclavian venipuncture in a patient who had previously undergone median sternotomy associated with coronary artery bypass grafting
Ray and Gupta^6^	1	Not described	Coronary bypass grafting
Sawalha and Gibbons^7^	1	Male	Bilateral lung transplantation performed through a transverse thoracosternotomy (“clamshell”) incision
Kwon et al^8^	1	Male	Following left-sided pneumonectomy
Sakamoto et al^9^	1	Male	Following Nuss Barr, surgical correction of a pectus excavatum**,** surgery
Engeler et al^10^	6	3 Male, 3 female	Heart lung transplantation
Rali and Manyam^11^	1	Female	Implantable cardioverter defibrillator placement, atrial lead micro-perforation, or a traumatic pleural puncture either leading to or in the presence of a buffalo chest
Lee et al^12^	1	Female	Heart-lung transplantation
Wittich et al^13^	3	2 Male, 1 female	Heart-lung transplantation, heart transplantation, Blalock-Taussig shunt placed by means of a right thoracotomy
Masuda and Ishida^14^	1	Male	Resection of an esophageal carcinoma
Kawakami and Namkoong^15^	1	Male	Esophagectomy
Johri et al^16^	1	Male	Thymoma resection
Paranjpe et al^17^	5	Not described	Heart-lung transplantation
Abd-Elsayed et al^18^	1	Male	Thymectomy
Groarke et al^19^	1	Male	Esophagectomy and substernal gastric interposition
Chan and Stark^20^	2	Both male	Both patients coronary artery bypass
Eguchi et al^21^	1	Male	Esophagectomy

The other 15 case reports mentioned 16 patients with no history of cardiothoracic surgery who developed simultaneous bilateral pneumothoraces.^22^, ^23^, ^24^, ^25^, ^26^, ^27^, ^28^, ^29^, ^30^, ^31^, ^32^, ^33^, ^34^, ^35^, ^36^ Six of these patients presented with spontaneous simultaneous bilateral pneumothoraces.^22^, ^23^, ^24^^,^^30^^,^^32^^,^^35^ In the other 10 cases, the pneumothoraces were due to an iatrogenic cause following an unilateral transbronchial biopsy,^25^, ^26^ transthoracic lung biopsy,^27^ chest tube thoracostomy,^28^ pacemaker placement,^29^ mechanical ventilation,^33^ or tracheostomy,^31^^,^^34^ or following central venous catheterization of the right subclavian vein^36^ in the absence of previous cardiothoracic surgery (Table 2).

**Table 2:** Simultaneous Bilateral Pneumothorax in Patients Without History of (Cardio)Thoracic Surgery

Authors	No. of Cases	Patient Sex	Iatrogenic Event
Parsons and Detterbeck^22^	1	Male	None
Jacobi et al^23^	1	Male	None
Hata et al^24^	1	Female	None
Hartin et al^25^	1	Female	Unilateral transbronchial biopsy
Findik et al^26^	1	Female	Unilateral transbronchial biopsy
Yamaura et al^27^	2	Both male	Transthoracic lung biopsy
Samuel and Mahmood^28^	1	Female	Chest-tube thoracostomy
Darwich and Tyrrell^29^	1	Female	Pacemaker placement
Bilavsky et al^30^	1	Male	None
Himeno and Tamura^31^	1	Male	Tracheostomy
Bassily-Marcus et al^32^	1	Female	Following mechanical ventilation
Albores et al^33^	1	Male	None
Kim and Kim^34^	1	Female	Following tracheostomy
Juvonen et al^35^	1	Female	None
Pazos et al^36^	1	Male	Following central venous catheterization of the right subclavian vein

Ever since the article by Schorlemmer et al^3^ was published, other authors used the term buffalo chest in case reports and textbooks when describing a bilateral pneumothorax.^1^^,^^4^, ^5^, ^6^, ^7^, ^8^, ^9^, ^10^, ^11^^,^^14^, ^15^, ^16^, ^17^^,^^19^, ^20^^,^^22^^,^^28^, ^29^, ^30^^,^^32^^,^^37^, ^38^, ^39^, ^40^ This was often accompanied by the anecdote of a Native American who was able to kill a bison with a single arrow or gunshot to the chest.^1^^,^^4^, ^5^, ^6^, ^7^^,^^11^^,^^16^^,^^19^, ^20^^,^^22^^,^^23^, ^24^, ^25^^,^^29^, ^30^^,^^38^, ^39^, ^40^ We questioned if this buffalo chest was just a myth or an actual anatomic anomaly in bison.

Because we could not find any literature on the anatomy of the mediastinum of the North American bison, we sent a questionnaire by e-mail to almost 50 Dutch veterinarians. No one could give us any information about communications of the pleural cavities in bison. We then consulted M. L. J Kik DVM, PhD, a veterinary pathologist specialized in exotic animals at the University of Utrecht in The Netherlands, and P. Cornillie, DVM, PhD, who specialized in veterinary anatomy at the University of Gent in Belgium. They contacted one colleague in Germany and two veterinarians in the United States, but no one could confirm that bison have fenestrations in their mediastinum. We then decided to search for proof of the existence of the “buffalo chest” in North American bison ourselves.

**Figure 1:**
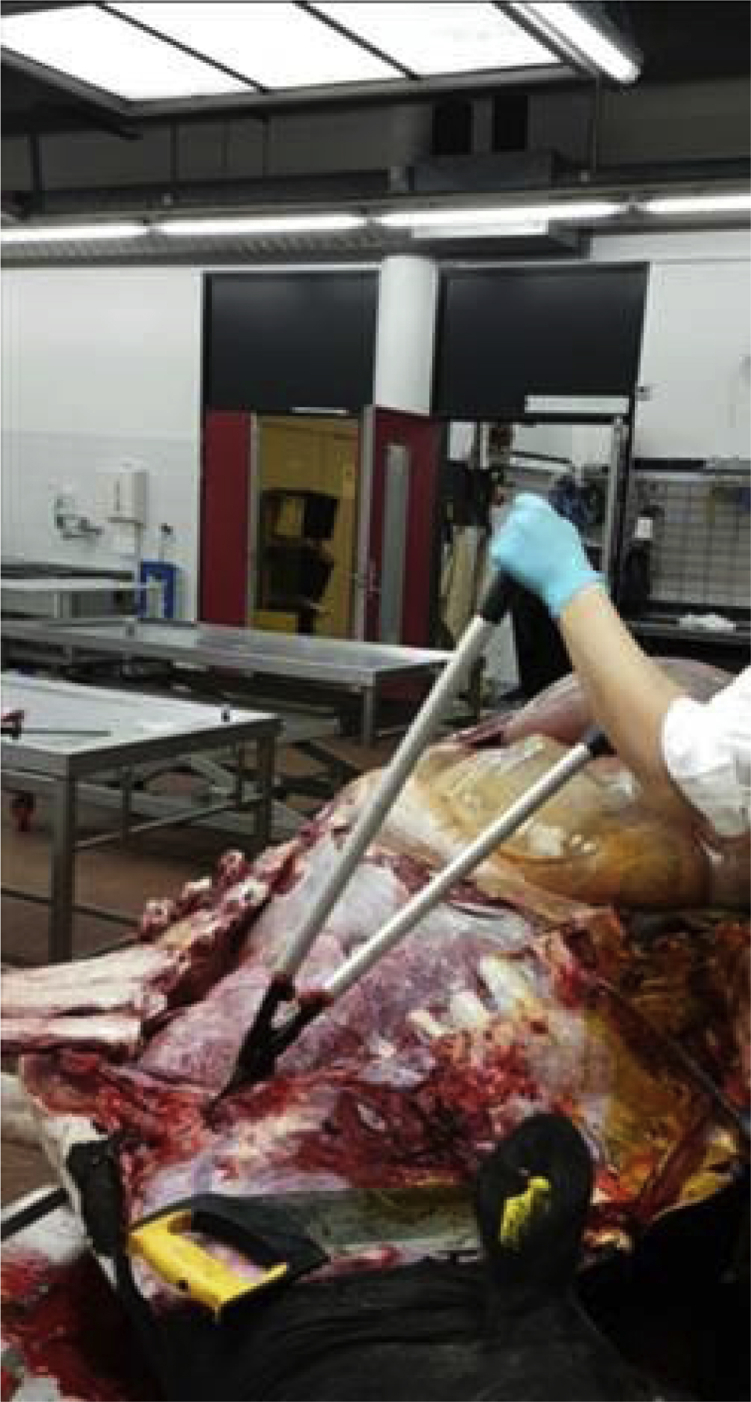
*Autopsy on a Dutch cow*.

We aimed to verify the actual presence of a connection between the two pleural cavities in North American bison. First, we were trained to perform autopsies on cows, which had died of natural causes, at the Faculty of Veterinary Medicine at the University of Utrecht (Fig 1). Together with Dr Kik and her colleague Louis van den Boom, DVM, we designed and tested three methods to investigate the existence of interpleural communications in the mediastinum of the North American bison.

The first method was a thorough visual examination of fenestrations of the anterior and posterior mediastinum of the North American bison following a thoracotomy. For the second method, we were inspired by the article of Hata et al.^24^ They described a case of a man with a simultaneous bilateral pneumothorax who was scheduled for a bullectomy via video-assisted thoracoscopy surgery. When they finished their surgery on the right lung and performed an air-leakage test with fluid, they noticed fluid production in the chest tube that was placed on the contralateral side.

We decided to name this method the “thoracic drainage system method,” a method in which a 32F chest tube is placed in each pleural cavity of the animal, and water with methylene blue would be instilled on one side. A digital thoracic drainage system with suction^41^ would be connected to the chest tube placed on the other side. If an interpleural communication existed, we would see drainage of blue water that was instilled in the contralateral pleural cavity.

For the third method, we inserted water with methylene blue in one pleural cavity after placing a chest tube on one side of the thorax. If the pleural cavity was full of water, we would open the pleural cavity of the contralateral side and perform a thorough visual examination of the pleura to investigate if water crossed the other side through an interpleural communication. The animal was laid on its back while performing this method.

## Results

**Figure 2:**
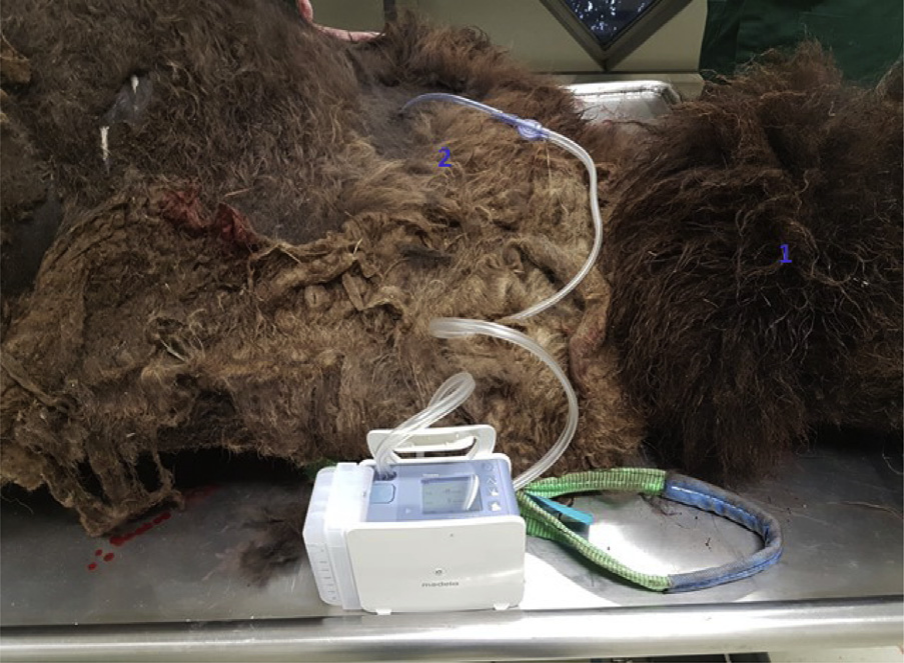
Failed drainage of fluid in the contralateral pleural cavity after suction with a digital drainage system. ^1^Cranial side of the animal. ^2^Chest tube present in the left pleural cavity.

**Figure 3:**
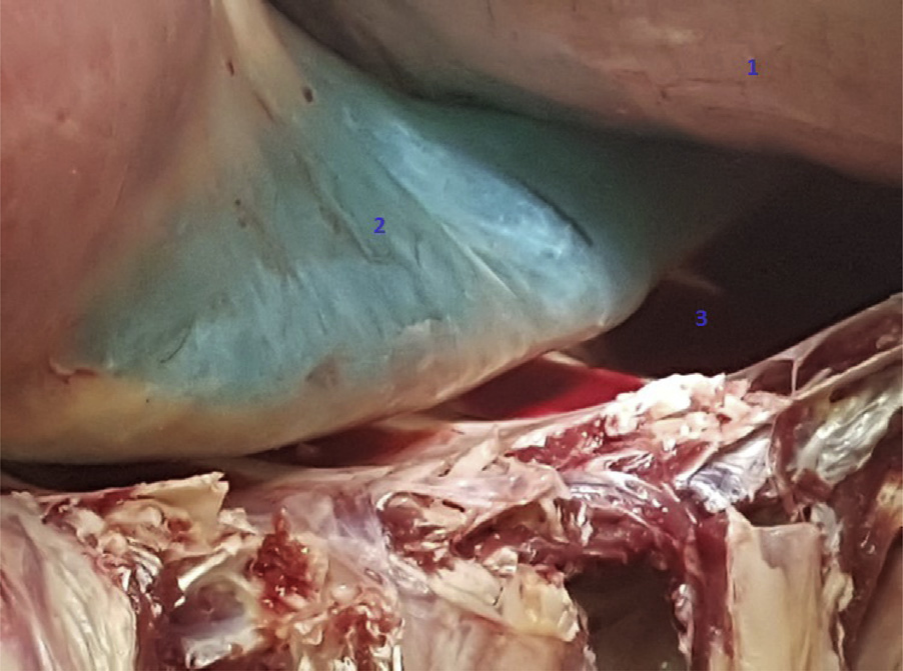
A closed mediastinum posterior: no leakage of methylene blue. ^1^Diaphragm. ^2^Posterior junction line. ^3^Left pleural cavity.

We were invited to the Rotterdam Zoo, Rotterdam, The Netherlands, to perform an autopsy on one of the North American bison that was euthanized for medical reasons other than respiratory problems. We started with placing chest tubes in both pleural cavities. After inserting almost 4 L of water with methylene blue in the right chest tube, we observed no drainage of water in the thoracic drainage system on the contralateral side of the pleural cavity (Fig 2). We continued with our third method by opening the contralateral pleural cavity and investigated the anterior and posterior mediastinum. We did not observe any blue water in the left cavity (Fig 3), indicating there was no sign of a buffalo chest.

Because there is only one small herd of nine North American bison in our country, we decided to go to the United States, where there are larger herds of North American bison, to further investigate the existence of the buffalo chest.

We first went to Benson, a small town in the south of Arizona, to visit Dr. Kenneth Throlson, the veterinarian who, through his “personal communication,” was the first to describe a buffalo chest.^3^ A large-animal veterinarian, he had years of experience working with thousands of North American bison. He confirmed to us, that during his work as a veterinarian, he noticed that a lot of bison died following a fight that resulted in penetration of horns unilaterally in the chest. Out of curiosity, he performed autopsies on these cases and found bilateral pneumothoraces and interpleural communications in the mediastinum in approximately one-half of these bison. Dr Throlson was surprised to learn that his anecdote about the North American bison had turned into “buffalo chest,” a well-established term for a clinical condition in humans.

Dr Throlson brought us in contact with the owner of a large meat-processing plant for North American bison in Brush, Colorado. We were only able to perform our first investigation method, due to the strict slaughter schedule and the fact that the meat could not be approved for human consumption after contamination with methylene blue.

**Figure 4:**
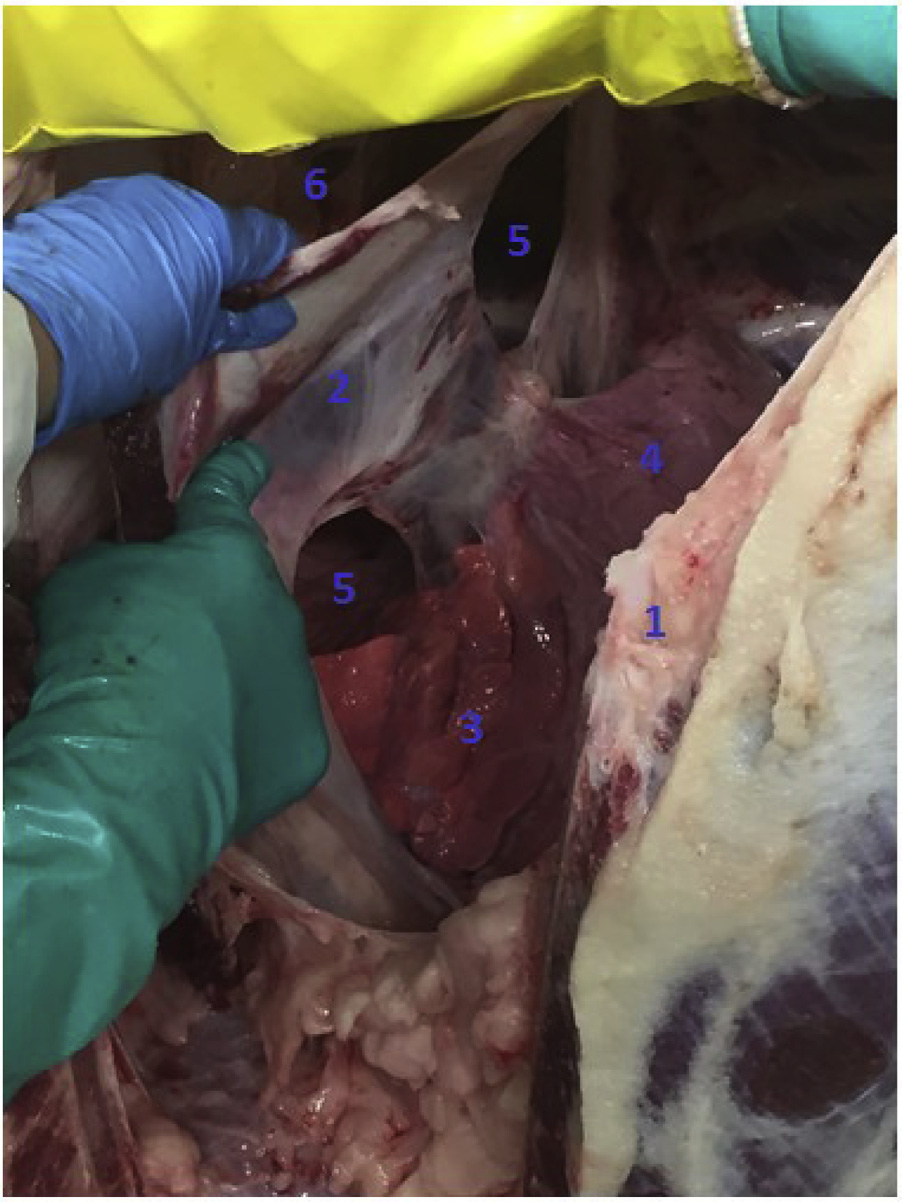
Fenestrations in the posterior mediastinum in a North American bison, with the animal placed in a head-down position. ^1^Sternum. ^2^Left pleura parietalis. ^3^Left lung. ^4^Heart. ^5^Fenestrations in posterior mediastinum. ^6^Pleural cavity.

**Figure 5:**
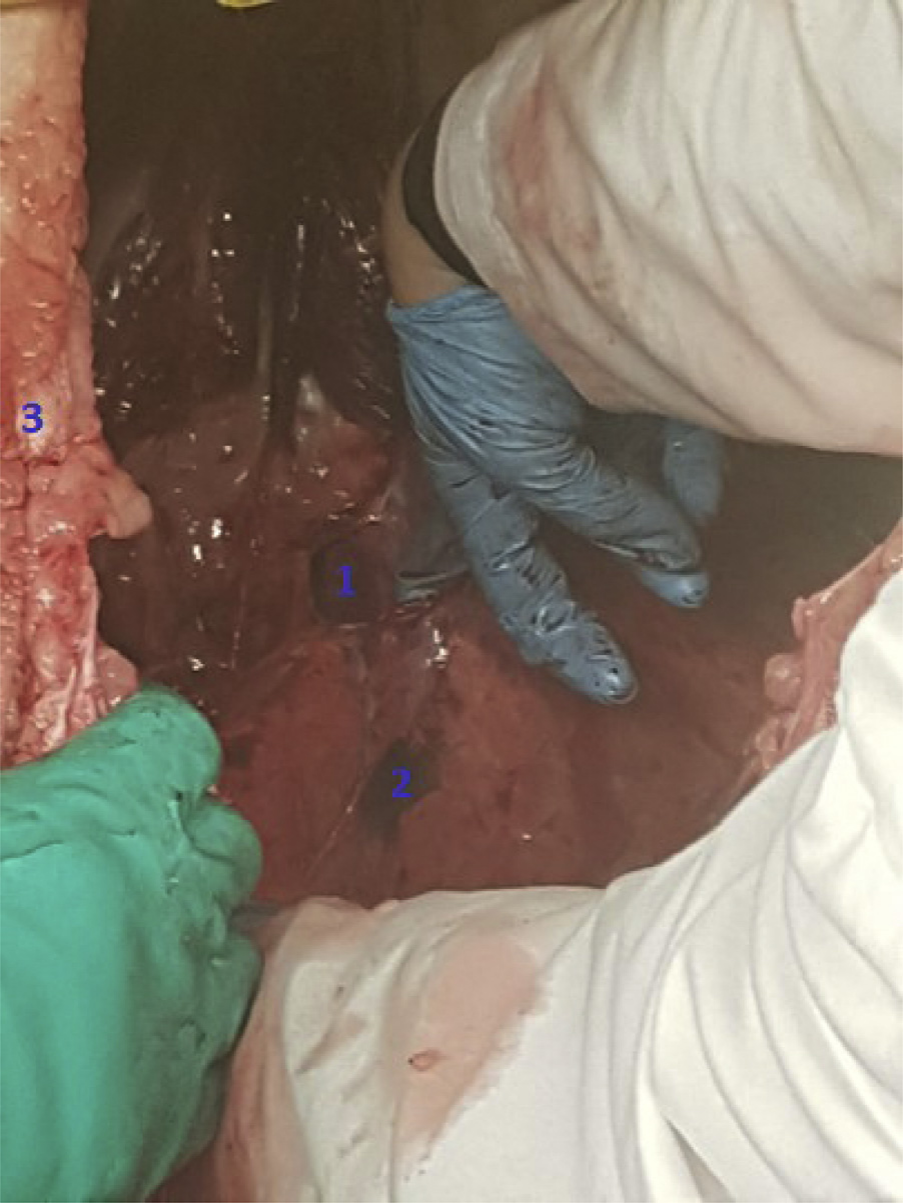
Fenestrations in the posterior mediastinum of a North American bison, with the animal in a head-down position with the lungs pushed aside. ^1^Fenestrations. ^2^Lung. ^3^Sternum.

Nevertheless, we were able to investigate the mediastinum of eight North American bison following thoracotomy. Data were collected with photography of the anterior and posterior mediastinum. Two of eight bison had visible fenestrations in their anterior mediastinum (Figs 4, 5), and two bison had visible fenestrations in both the anterior and posterior mediastinum; the other four bison had no visible intrapleural fenestrations.

## Discussion

Just like in some other mammal species and the North American bison, an idiopathic cause of interpleural communication can occur in humans. Although some articles state this action must be caused by a congenital abnormality,^3^^,^^5^, ^6^, ^7^^,^^11^^,^^16^^,^^20^, ^21^, ^22^, ^23^, ^24^, ^25^^,^^27^, ^28^, ^29^, ^30^^,^^32^, ^33^^,^^35^^,^^37^, ^38^, ^39^, ^40^ little is known about the embryologic development of the lungs and pleurae. Most of our knowledge on human development is based on the work of early embryologists, often published more than a century ago, and is established on embryologic studies in mouse models.^42^ The pleural, peritoneal, and pericardial cavities all derive from the intra-embryonic coelom. This primitive body cavity develops in the primitive mesodermal layer of the embryo.^43^ Closure of the pericardial-peritoneal canals starts during Carnegie stage 12 (26-30 days) and is completed in stage 18 (44-48 days). Between these stages, the mesenchymal ridge grows bilaterally into the pericardial-peritoneal canals until they end up as two separate cavities: the pericardial cavity and the pleural cavity.^44^ Norden et al^45^ found that mice lacking the Tbox-18 gene did not form this mesenchymal ridge; they also found that the Wilms tumor-1 (Wt1) gene, a zinc finger transcription factor expressed in certain mesoderm-derived tissues as the pleura,^46^ plays a role in the closure of the pericardioperitoneal canals. De Bakker et al,^44^ who reconstructed the embryos of the mice used by Norden et al^45^ into three-dimensional images, support this finding. Lack of these genes in humans could be a possible explanation for a congenital buffalo chest.

In *Thurlbeck’s Pathology of the Lung,* it is stated that apoptosis has been observed in all stages of lung development.^42^ Although its role and regulation are not well understood, apoptosis probably is the key player in fetal lung development. This could also be a plausible explanation for a congenital buffalo chest. Jacobi et al^23^ hypothesized that disruption of the mediastinal pleura from lung metastasis is another possible explanation for developing an interpleural communication.

There are a number of limitations of this study that deserve mention. The study population was small, and we could not perform every method on each North American bison due to strict rules that apply to safe meat processing. It is possible that small fenestrations in both the anterior and posterior mediastinum were overlooked with the first method. These could have been better detected by using the second and third method, which are considered to be more accurate than the first.

For ethical reasons, we decided not to kill bison solely for investigating the mediastinum. This study is important because it brings attention to a condition that was solely based on an anecdote without physical evidence. This evidence was then included in a scientific study. To our knowledge, this study is the first to prove the existence of the buffalo chest.

Fenestrations in the mediastinum have also been noted in dogs, horses, cats, and rabbits.^47^, ^48^, ^49^, ^50^, ^51^, ^52^, ^53^ For example, in a study of induced pneumothorax in 22 of 24 dogs in which air was injected into one pleural space, these dogs quickly developed bilateral pneumothorax.^47^

Human share many similarities with other mammals in embryologic development. Even though only a few cases of a buffalo chest in humans have been described, we presume that the incidence of interpleural communication in humans is underestimated because it can only be diagnosed if a pneumothorax occurs, or during autopsy if the pathologist focuses on the thorax anatomy regarding this condition. It is therefore important to always pay attention to the contralateral chest when a patient presents with a pneumothorax.

The presence of an interpleural communication can be advantageous. At least in theory, bilateral pneumothorax or effusions in the presence of an interpleural communication could be treated with just one chest tube, thereby avoiding the need for and risks of an additional chest tube on the contralateral side.^3^^,^^6^^,^^10^^,^^13^^,^^17^^,^^23^^,^^25^ It should be noted, however, that in one case this approach failed and actually progressed to a contralateral tension pneumothorax.^12^

The presence of an interpleural communication also involves a potential disadvantage. In case of a pneumothorax, there is a realistic risk of a contralateral tension pneumothorax at the undrained side, when the interpleural communication functions as a one-way valve. It is important to be aware of this potential risk and to always consider it if a a patient who received a chest tube deteriorates further.

## Interpretation

Many authors have used the term “buffalo chest” when describing a bilateral pneumothorax due to interpleural communications, and many clinicians may be familiar with this condition in humans. Hence, it is important to always examine the contralateral lung when a patient presents with a pneumothorax, because missing a bilateral pneumothorax could have fatal consequences as it can result in hemodynamic deterioration due to a tension pneumothorax**.**

## References

[1] Feller-Kopman D., Light R. (2018). Pleural disease. N Engl J Med.

[2] Sayar A., Turna A., Metin M., Kucukyagci N., Solak O., Gurses A. (2004). Simultaneous bilateral spontaneous pneumothorax report of 12 cases and review of the literature. Acta Chir Belg.

[3] Schorlemmer G.R., Khouri R.K., Murray G.F., Johnson G. (1984). Bilateral pneumothoraces secondary to iatrogenic buffalo chest. Ann Surg.

[4] Grathwohl K.W., Derdak S. (2003). Images in clinical medicine. Buffalo chest. N Engl J Med.

[5] Ikezoe K., Tanaka E., Tanizawa K. (2012). Bilateral dissemination of malignant pleural mesothelioma via iatrogenic buffalo chest: a rare outcome of disease progression. Gen Thorac Surg.

[6] Ray A., Gupta M. (2017). Iatrogenic buffalo-chest syndrome. Indian J Radiol Imaging.

[7] Sawalha L., Gibbons W.J. (2015). Iatrogenic “buffalo chest” bilateral pneumothoraces following unilateral transbronchial lung biopsies in a bilateral lung transplant. Respir Mede C Rep.

[8] Kwon J.B., Moon S.W., Park C.B. (2012). Spontaneous pneumothorax in a patient with buffalo chest. Eur J Cardiothorac Surg.

[9] Sakamoto K., Ando K., Noma D. (2014). Spontaneous bilateral pneumothorax resulting from iatrogenic buffalo chest after the Nuss procedure. Ann Thorac Surg.

[10] Engeler C.E., Olson P.N., Engeler C.M. (1992). Shifting pneumothorax after heart-lung transplantation. Radiology.

[11] Rali A.S., Manyam H. (2015). Bilateral pneumothoraces following BiV ICD placement: a case of buffalo chest syndrome. Am J Case Rep.

[12] Lee Y.C., McGrath G.B., Chin W.S., Light R.W. (1999). Contralateral tension pneumothorax following unilateral chest tube drainage of bilateral pneumothoraces in a heart-lung transplant patient. Chest.

[13] Wittich G.R., Kusnick C.A., Starnes V.A., Lucas D.E. (1992). Communication between the two pleural cavities after major cardiothoracic surgery: relevance to percutaneous intervention. Radiology.

[14] Masuda T., Ishida J. (2020). Simultaneous bilateral pneumothorax due to communication of both thoracic cavity. Jpn Jl Thorac Surg.

[15] Kawakami N., Namkoong H. (2019). Buffalo chest syndrome following esophagectomy. Intern Med.

[16] Johri S., Berlin D., Sanders A. (2003). Bilateral pneumothoraces after unilateral transthoracic needle biopsy of a lung nodule. Chest.

[17] Paranjpe D.V., Wittich G.R., Hamid L.W., Bergin C.J. (1994). Frequency and management of pneumothoraces in heart-lung transplant recipients. Radiology.

[18] Abd-Elsayed A.A., Ghaly T., Farag E., Esa W.A.S. (2013). Bilateral pneumothoraces following a right subclavian catheter insertion after thymectomy for a patient with a myasthenic crisis. Ochsner J.

[19] Groarke J., Breen D., O’Connell F., O’Donnell R. (2007). Bilateral pneumothorax resulting from a diagnostic thoracentesis. Eur Respir J.

[20] Chan F., Stark P. (1997). Bilateral pneumothoraces after coronary bypass surgery—a case of ‘buffalo chest. Clin Intensive Care.

[21] Eguchi T., Hamanaka K., Kobayashi N. (2011). Occurrence of a simultaneous bilateral spontaneous pneumothorax due to a pleuro-pleural communication. Ann Thorac Surg.

[22] Parsons A.M., Detterbeck F.C. (2005). Of buffaloes, horseshoes, and having no connections. Ann Thorac Surg.

[23] Jacobi A., Eber C., Weinberger A., Friedman S.N. (2016). Bilateral pneumothoraces after unilateral lung biopsy—a case of “buffalo chest.”. Am J Respir Crit Care Med.

[24] Hata Y., Suzuki T., Yokoi M. (2013). Simultaneous bilateral spontaneous pneumothorax with congenital pleuro-pleural communication. J Thorac Dis.

[25] Hartin D.J., Kendall R., Boyle A.A., Atkinson P.R.T. (2006). Case of the month: buffalo chest: a case of bilateral pneumothoraces due to pleuropleural communication. Emerg Med J.

[26] Findik S., Erkan L., Light R.W. (2006). Iatrogenic bilateral pneumothorax following unilateral transbronchial lung biopsy. Br J Radiol.

[27] Yamaura H., Inaba Y., Sato Y., Najima M., Shimamoto H., Nishiofuku H. (2007). Bilateral pneumothorax after unilateral transthoracic puncture. J Vasc Interv Radiol.

[28] Samuel A., Mahmood N. (2020). Buffalo chest-tube thoracostomy gone too far: a case report. Am J Respir Crit Care Med.

[29] Darwich N.S., Tyrrell R.L. (2019). Bilateral pneumothorax after pacemaker placement “buffalo chest.”. Respir Med Case Rep.

[30] Bilavsky E., Yarden-Bilavsky H., Waisman Y., Marcus N. (2008). Bilateral primary spontaneous pneumothorax: buffalo chest. Pediatr Emerg Care.

[31] Himeno A., Tamura A. (2015). Bilateral pneumothoraces secondary to a Buffalo chest. Aust Crit Care.

[32] Bassily-Marcus A.M., Oropello J., Shaik A. (2011). Shifting pneumothorax. ICU Director.

[33] Albores J., Abtin F., Barjaktarevic I. (2015). A 44-year-old man with bilateral pneumothorax. Chest.

[34] Kim W.H., Kim B.H. (2012). Bilateral pneumothoraces, pneumomediastinum, pneumoperitoneum, pneumoretroperitoneum, and subcutaneous emphysema after percutaneous tracheostomy—a case report. Korean J Anesthesiol.

[35] Juvonen T., Lepojarvi M., Pokela R., Juvonen J., Kairaluoma M.I. (1992). Mediastinal window: a cause of simultaneous bilateral spontaneous pneumothorax. Ann Thorac Surg.

[36] Pazos F., Masterson K., Inan C., Robert J., Walder B. (2009). Bilateral pneumothoraces following central venous cannulation. Case Report Med.

[37] Hiremath S., Hegde H.V., Swamy P.R.S., Bhat Pai R. (2012). Tension pneumothorax and pneumomediastinum caused by a malpositioned mediastinal drain in a patient following closure of an atrial septal defect. J Cardiothorac Vasc Anesth.

[38] Verma N., Knight B.P. (2019). Update in cardiac pacing. Arrhythm Electrophysiol Rev.

[39] Lehmann V., Keller W., Egger B. (2020). Systematic review of pneumothoraces after endoscopic retrograde cholangiopancreatography. Swiss Med Wkly.

[40] Preston R.R., Wilson T.E. (2013).

[41] (2011). Thopaz Digital Chest Drainage System.

[42] Chung A.M., Meyers J.L., Tazelaar H.D. (2005).

[43] Light R.W., Lee Y.C.G. (2016).

[44] De Bakker B.S., de Jong H., Hagoort J. (2016). An interactive three-dimensional digital atlas and quantitative database of human development. Science.

[45] Norden J., Grieskamp T., Kispert A., Moorman A., Christoffels V., Englert C. (2012). Partial absence of pleuropericardial membranes in Tbx18- and Wt1-deficient mice. PLoS One.

[46] Haber D.A., Buckler A.J., Glaser T. (1990). An internal deletion within an 11p13 zinc finger gene contributes to the development of Wilms tumor. Cell.

[47] Kern D.A., Carrig C.B., Martin R.A. (1994). Radiographic evaluation of induced pneumothorax in the dog. Vet Radiol Ultrasound.

[48] Gendron K., McDonough S.P., Flanders J.A., Tse M., Scrivani P.V. (2018). The pathogenesis of paraesophageal empyema in dogs and constancy of radiographic and computed tomography signs are linked to involvement of the mediastinal serous cavity. Vet Radiol Ultrasound.

[49] Canola P.A., Valadao C.A.A., Canola J.C., Flores F.N., Lopes M.C.S. (2019). Experimentally induced open pneumothorax in horses. Equine Vet Sci.

[50] Sprayberry K.A., Barrett E.J. (2015). Thoracic trauma in horses. Vet Clin Equine Prac.

[51] Boy M.G., Sweeney C.R. (2000). Pneumothorax in horses: 40 cases (1980-1997). Am Vet Med Assoc.

[52] Knecht C.D. (1973). Surgical treatment of diseases of the pleura, mediastinum and thoracic cage. Veterinary Surgery.

[53] Macklin C.C. (1937). Pneumothorax with massive collapse from experimental local over-inflation of the lung substance. Can Med Assoc J.

